# Supplementary material for: Consecutive multimaterial printing of biomimetic ionic hydrogel power sources with high flexibility and stretchability
Source: Nat Commun. 2024 Jun 19;15:5261. doi: 10.1038/s41467-024-49469-6 (PMC11187209; doi:10.1038/s41467-024-49469-6)
Supplement: Supplementary file 1 — Supplementary Information [file 41467_2024_49469_MOESM1_ESM.pdf]

Supplementary Information

Consecutive Multimaterial Printing of Biomimetic Ionic Hydrogel Power Sources with High Flexibility and Stretchability

Pei He[1,2], Junyu Yue[1,2], Zhennan Qiu[1,2], Zijie Meng[1,2,3], Jiankang He[1,2 *] and Dichen Li[1,2]

[1]State Key Laboratory for Manufacturing Systems Engineering, Xi'an Jiaotong University, Xi'an, 710049, China

[2]NMPA Key Laboratory for Research and Evaluation of Additive Manufacturing Medical Devices, Xi'an Jiaotong University, Xi'an, Shaanxi 710049, China

[3]Frontier Institute of Science and Technology, Xi'an Jiaotong University, Xi'an, 710049, China

*Corresponding author: jiankanghe@mail.xjtu.edu.cn

This PDF file includes:

Supplementary Table 1
Supplementary Figure 1 to 17

Other Supplementary Materials for this manuscript include the following:

Supplementary Movie 1-10

Supplementary Tables

Ink	HS ink	LS ink	CS ink	AS ink
Viscosity (mPa s^{-1})	2.21 ± 0.05	2.73 ± 0.11	2.16 ± 0.02	2.42 ± 0.05

Supplementary Table 1. The viscosities of four types of ionic hydrogel precursor solutions at 20 ℃.

Supplementary Figures

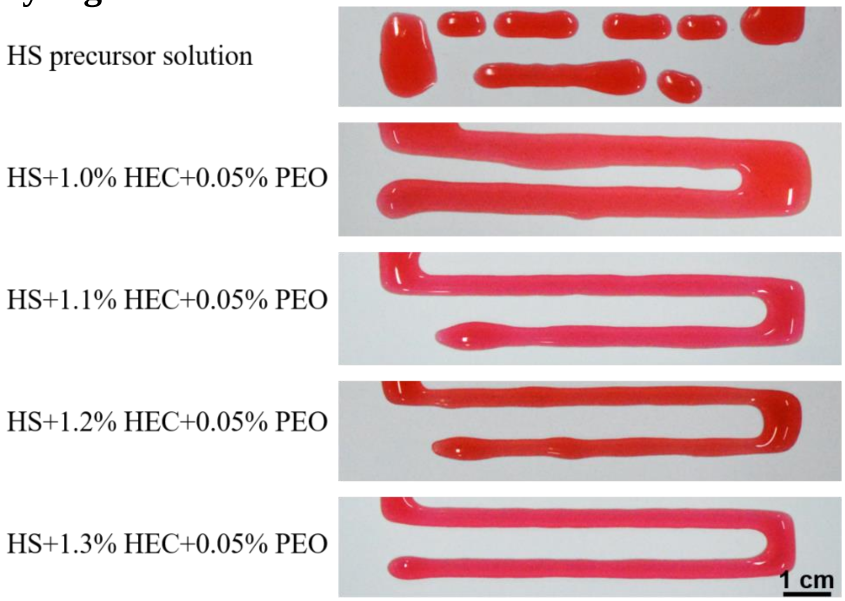

Supplementary Figure 1. The photograph of printed HS hydrogel filaments with different concentrations of HEC ranging from1.0% to1.3% (w/v %) at an interval of 0.1 and 0.05% PEO at the air pressure of 120 kPa.

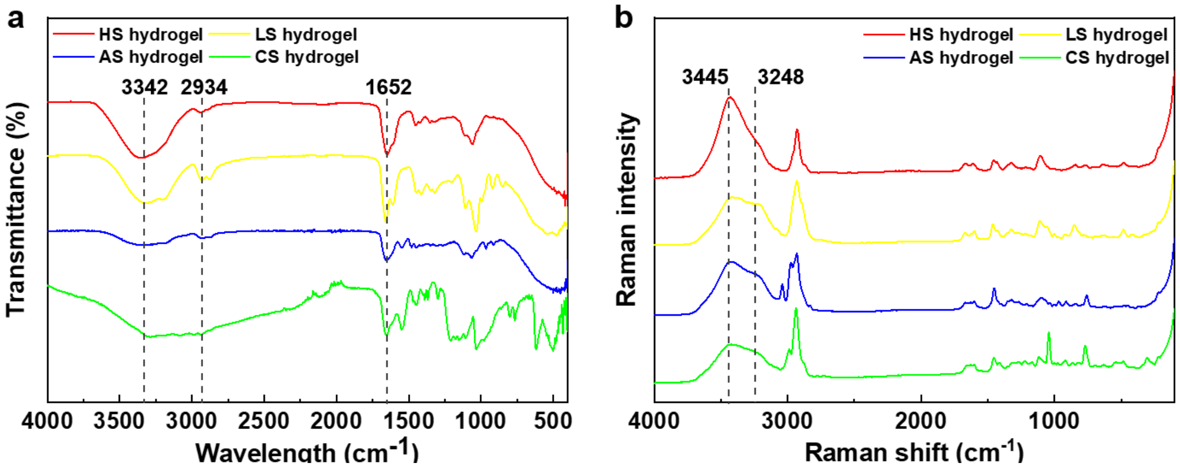

Supplementary Figure 2. (a) FTIR spectra of HS, LS, AS, and CS hydrogels characterized by using a Fourier transform infrared spectrometer (Nicolet iS10, Thermo Fisher, US) operating at a resolution of 1 $cm^{-1}$and (b) Raman spectra of HS, LS, AS, and CS hydrogels recorded by a Raman Spectroscopy system (LabRAM HR Evolution, HORIBA, Japan) with a 532 nm laser from 100 to 4000 cm^{-1} at a resolution of 0.2 cm^{-1}.

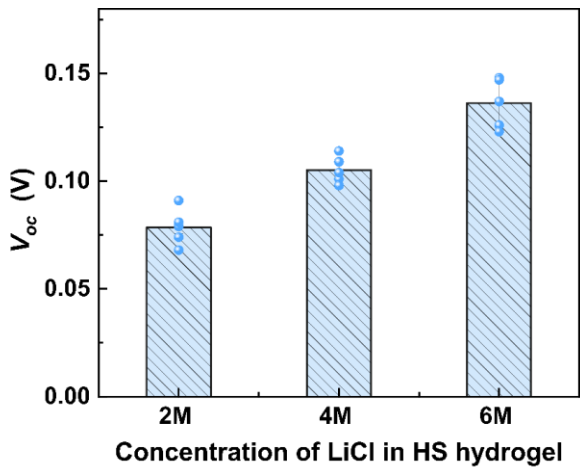

Supplementary Figure 3. The voltage (mean ± s.d., $n = 3$) of IHPS unit with a concentration of LiCl of 2.0 mol L^{-1}, 4.0 mol L^{-1}, and 6.0 mol L^{-1} in the HS hydrogel.

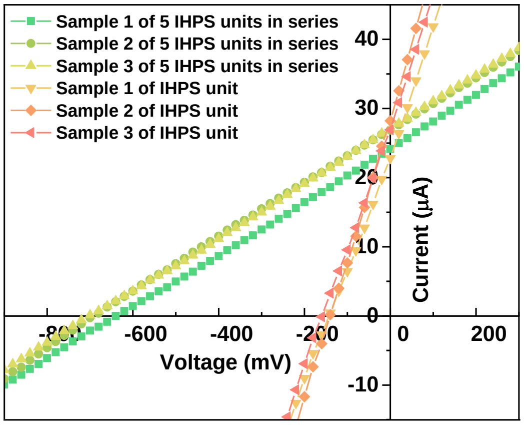

Supplementary Figure 4. The IV curves of consecutively-printed ionic hydrogel power source with a single unit and 5 units in series by using an electrometer (Keithley 6517b, America) with the applied voltage ranging from -1 V to 1 V with a step voltage of 0.02 V.

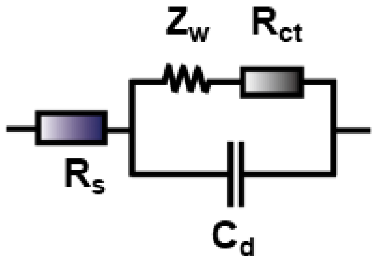

Supplementary Figure 5. The fitted equivalent circuit of consecutive IHPS unit and microfluidic perfused IHPS unit with stacked structure, where R_s refers to internal resistance of the IHPS unit, R_{ct} refers to charge transfer resistance, C_d refers to double layer capacitance, Z_W refers to and Warburg impedance.

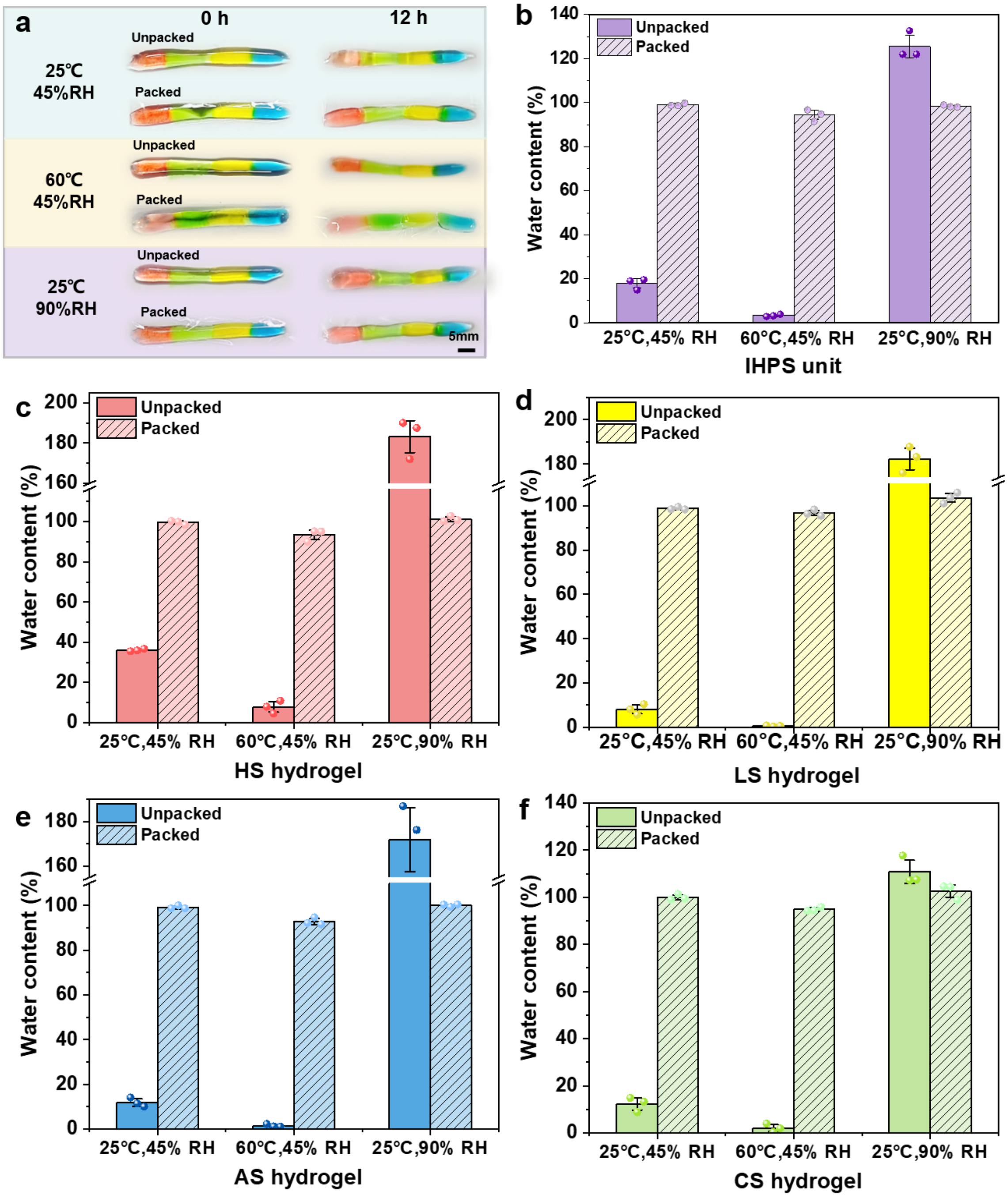

Supplementary Figure 6. Water retention tests of packed and unpacked consecutive IHPS units and four types of hydrogel materials. (a) Photographs and (b) water content (mean ± s.d., $n = 3$) of packed and unpacked IHPS unit stored at 25 °C and 45% RH, 60 °C and 45% RH, 25 °C and 90% RH for 0 and 12 h. Water content (mean ± s.d., $n = 3$) of packed and unpacked (c)HS, (d) LS, (e) AS and (f) CS hydrogel filament stored at 25 °C and 45% RH, 60 °C and 45% RH, 25 °C and 90% RH for 12 h.

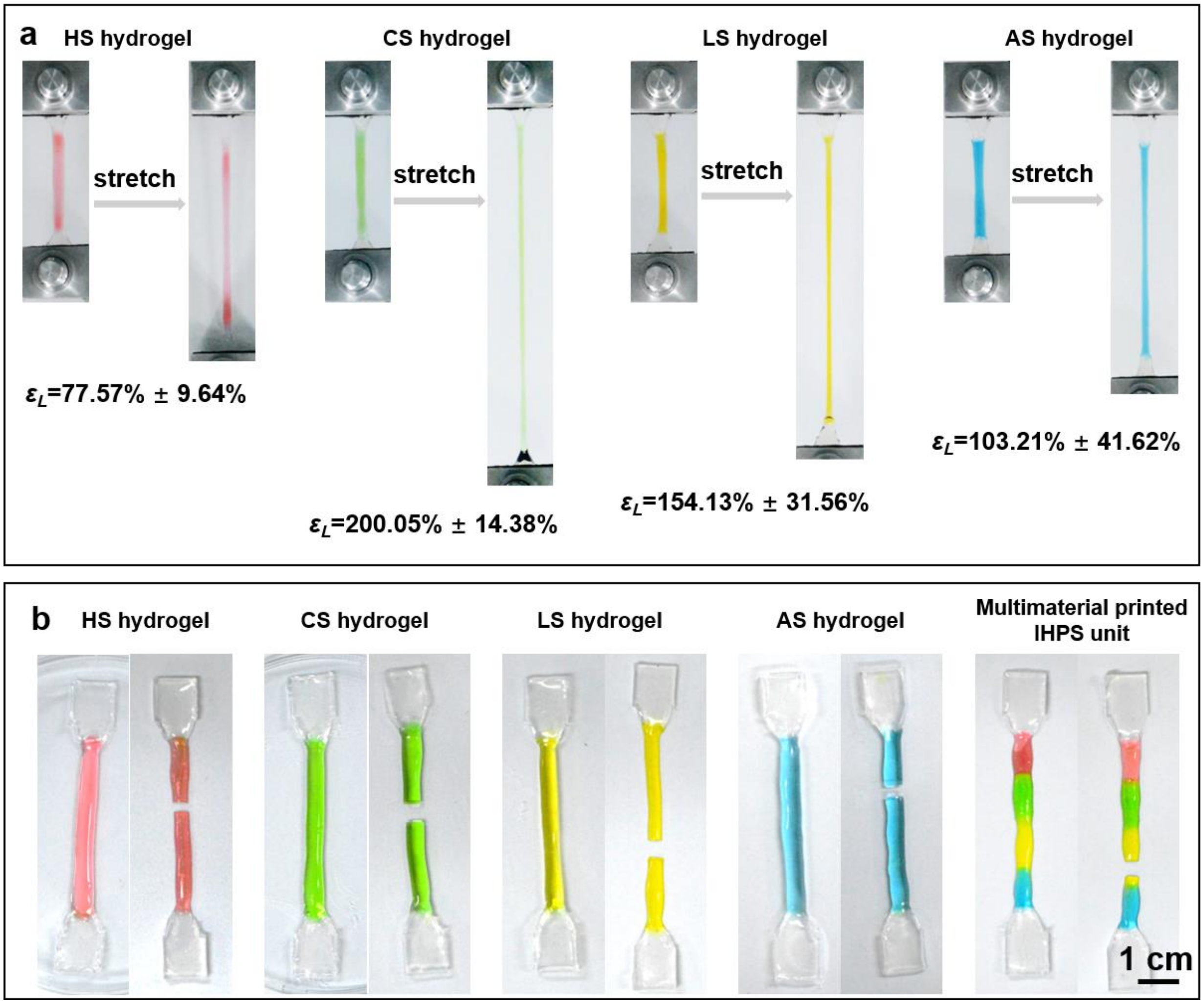

Supplementary Figure 7. (a) Photograph of mechanical test of the four ionic hydrogels. (b) Photographs of four types of hydrogels and consecutive IHPS unit pieces and that after fractured. Scale bar = 10 mm.

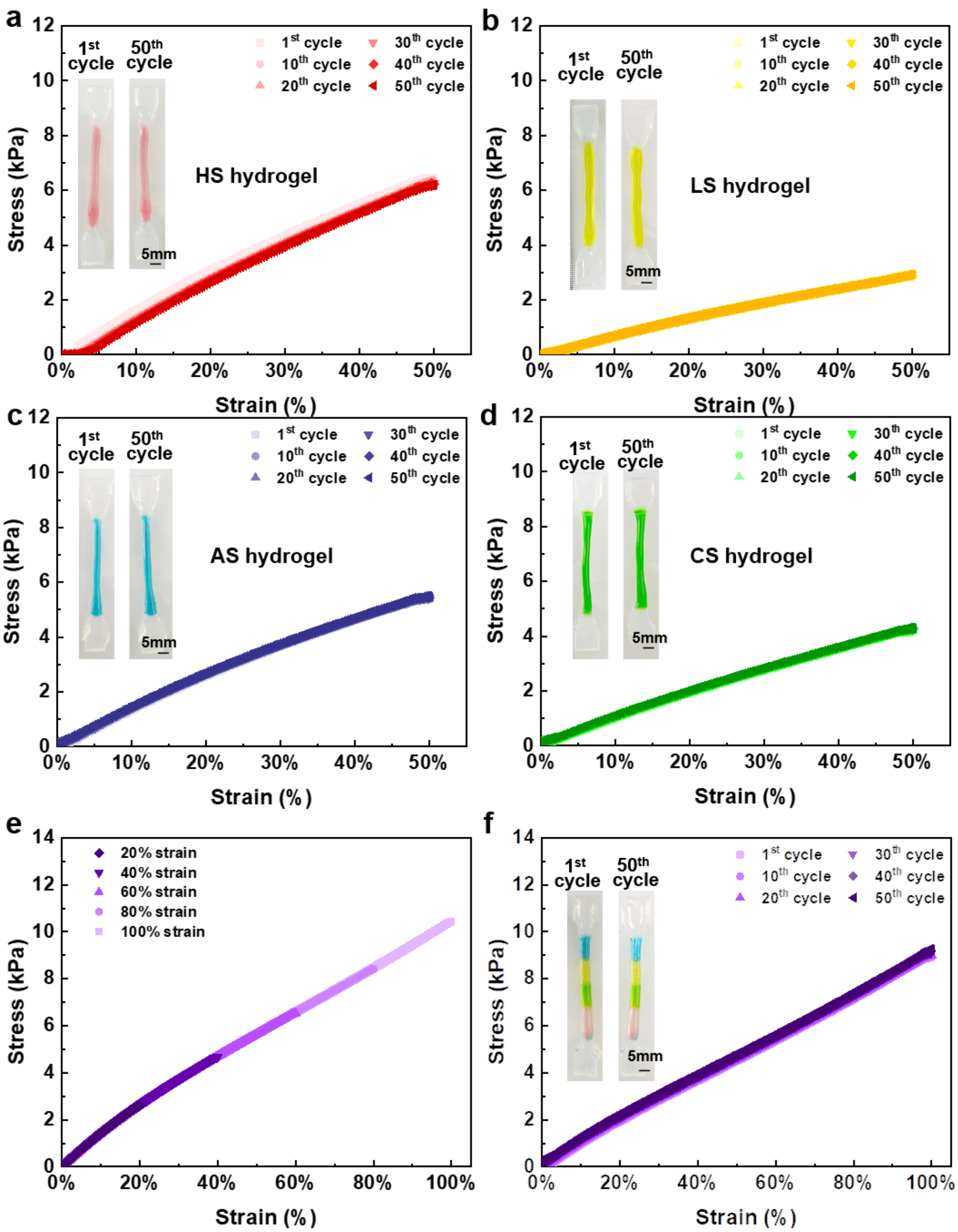

Supplementary Figure 8. Cyclic tensile tests of the four hydrogel samples and the consecutive IHPS unit. Cyclic tensile stress-strain curves of (a) HS hydrogel, (b) LS hydrogel, (c) AS hydrogel, and (d) CS hydrogel under a strain of 50% for 50 cycles. Insets show images of the four types of hydrogel samples before and after 50 stretching cycles under a strain of 50%. (e) Tensile stress-strain curves of IHPS unit at a strain of 20%, 40%, 60%, 80% and 100%, respectively. (f) Cyclic tensile stress-strain curves of IHPS unit under a strain of 100% for 50 cycles. Insets show images of the IHPS unit before and after 50 stretching cycles.

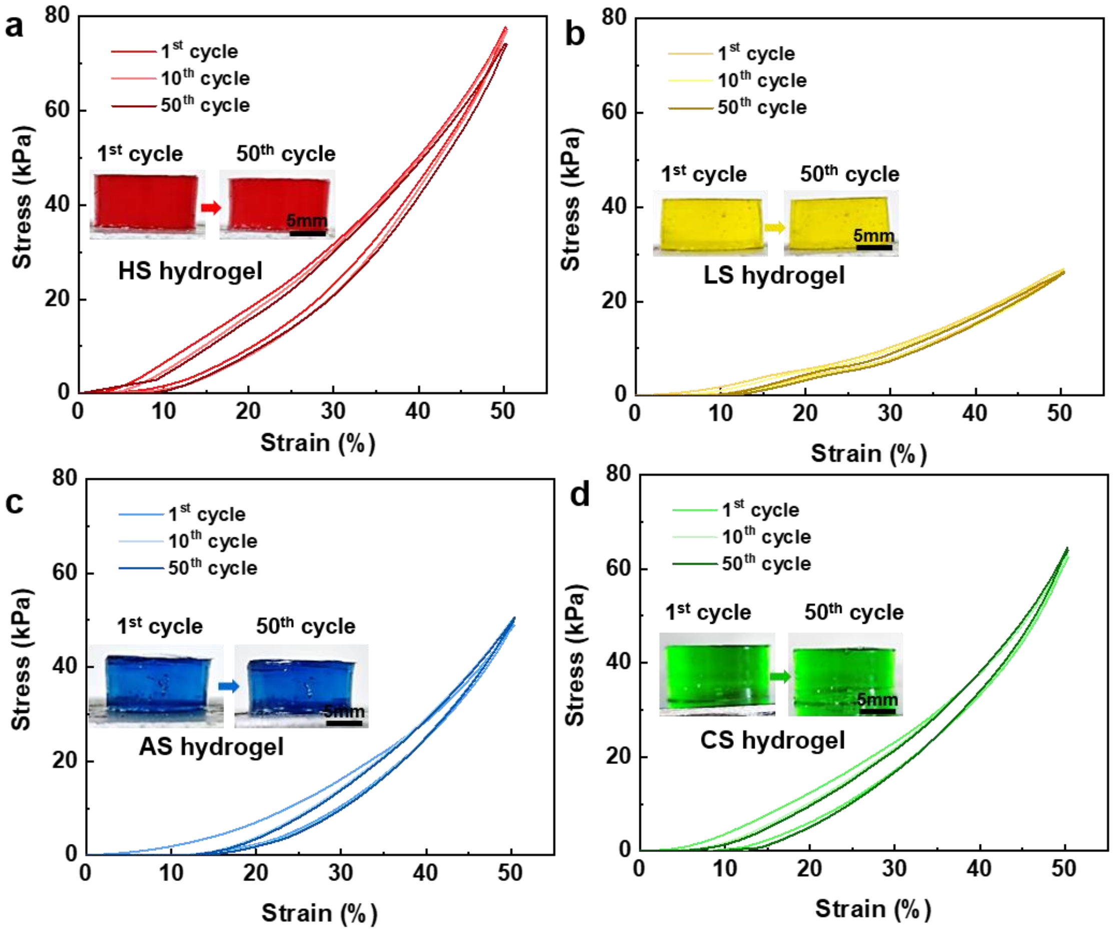

Supplementary Figure 9. Cyclic compressive tests of the four hydrogel materials. Cyclic compressive stress-strain curves of (a) HS hydrogel, (b) LS hydrogel, (c) AS hydrogel, and (d) CS hydrogel under a strain of 50% for 50 cycles. Insets show images of the four hydrogel samples before and after 50 compression cycles.

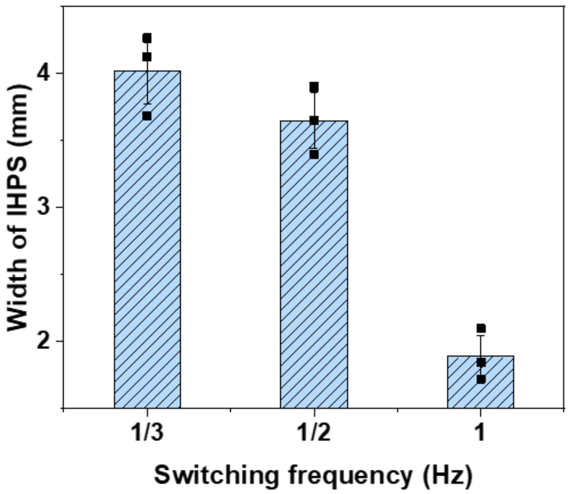

Supplementary Figure 10. The width (mean ± s.d., $n = 3$) of multimaterial printed IHPS filaments when the switching frequency varied from 1/3 Hz to 1 Hz while the air pressure, moving speed, and nozzle-to-collect distance were fixed at 140 kPa, 4.0 mm s^{-1}, and 5.0 mm respectively.

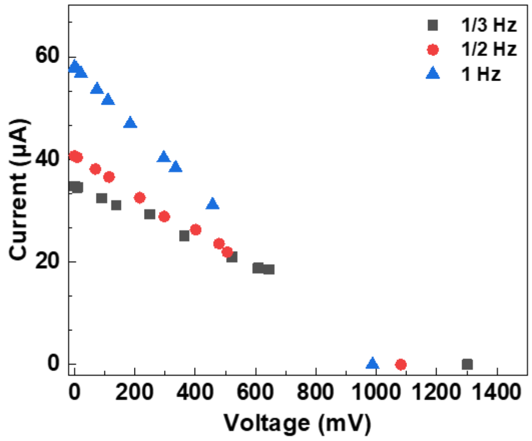

Supplementary Figure 11. Current and voltage curve of IHPS with 10 units in series at the switching frequency of 1/3 Hz, 1/2 Hz and 1 Hz.

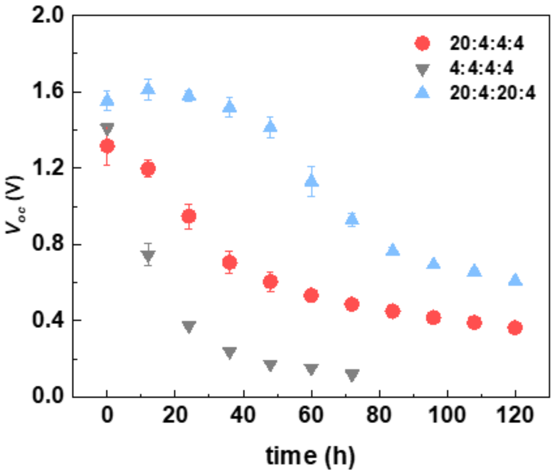

Supplementary Figure 12. Power dissipation profile of IHPS with 10 units in series with a proportion of 20:4:4:4 (mean ± s.d., $n = 3$) compared with that of 4:4:4:4 and 20:4:20:4.

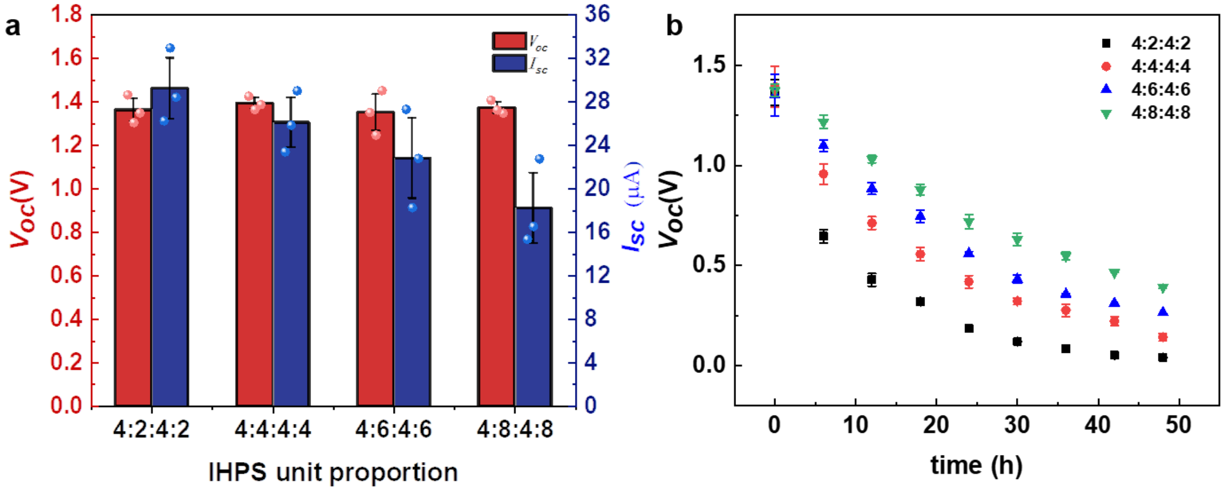

Supplementary Figure 13. V_{oc}, I_{sc}, and power dissipation (mean ± s.d., $n = 3$) of consecutively-printed 10-units IHPS with the HS:CS:LS:AS proportion of 4:2:4:2, 4:4:4:4, 4:6:4:6, and 4:8:4:8.

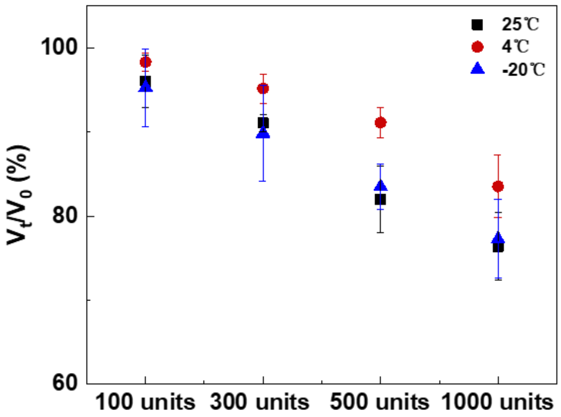

Supplementary Figure 14. The voltage (mean ± s.d., $n = 3$) of consecutively- printed IHPS with 10 units in series at the time point of finishing printing IHPS with 100, 300, 500 and 1000 units when incubated at the temperature of 25 °C, 4 °C and -20 °C.

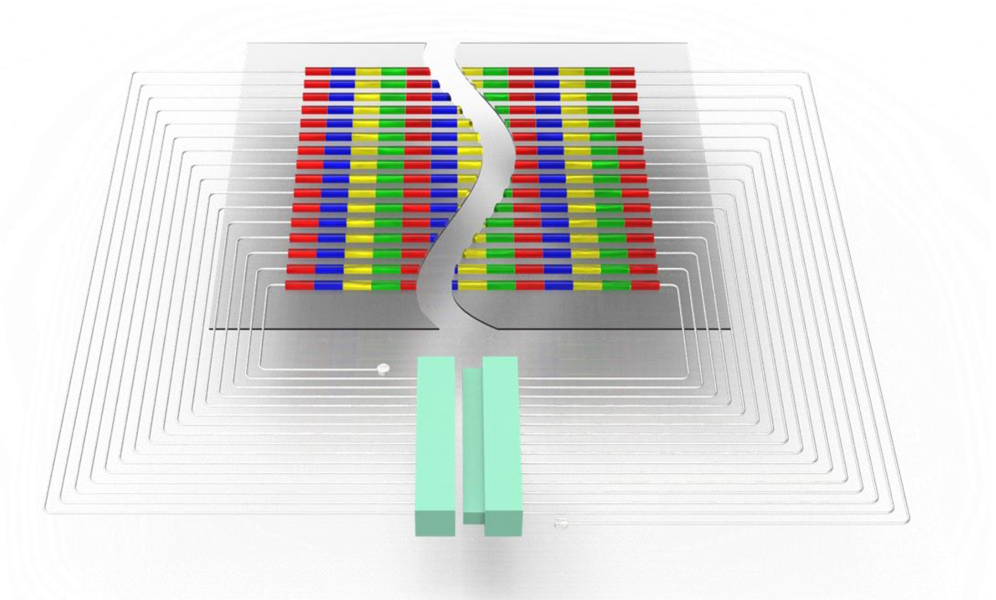

Supplementary Figure 15. Schematics of designed circuit for the multimaterial printed IHPS with arrayed multimaterial printhead for high voltage by connecting the adjacent battery positively and negatively via multichannel terminals.

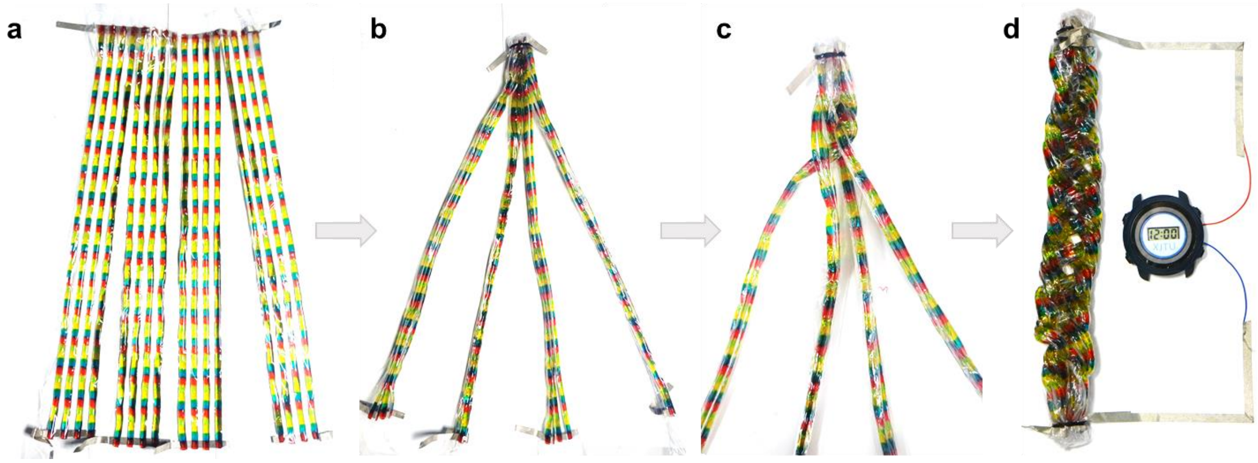

Supplementary Figure 16. Photograph of the process of compiling a 16-arrayed IHPS into a watch band.

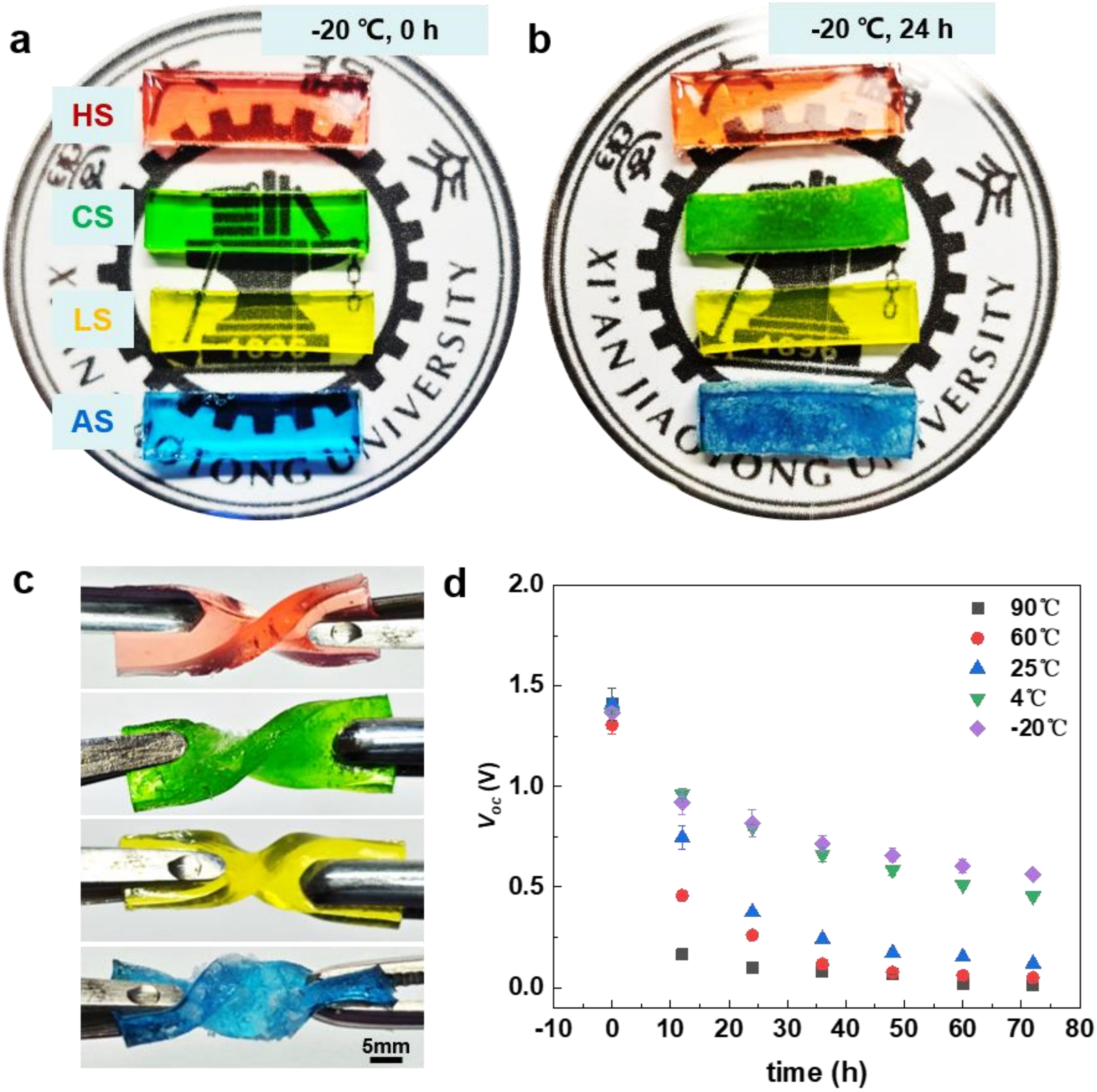

Supplementary Figure 17. Anti-freezing tests for the four types of hydrogel materials. (a) The transparency of four types of hydrogel materials at room temperature and (b) at -20 ℃ for 24 h. (c) The flexibility of four types of hydrogel materials after storage at -20 ℃ for 24 h. (d) Power dissipation (mean ± s.d., $n = 3$) of IHPS with 10 units in series at -20 ℃, 4 ℃, 25 ℃, 60 ℃ and 90 ℃.
