# Supplementary material for: Consecutive multimaterial printing of biomimetic ionic hydrogel power sources with high flexibility and stretchability
Source: Nat Commun. 2024 Jun 19;15:5261. doi: 10.1038/s41467-024-49469-6 (PMC11187209; doi:10.1038/s41467-024-49469-6)
Supplement: Supplementary file 3 — Description of Additional Supplementary Files [file 41467_2024_49469_MOESM3_ESM.pdf]

Description of Additional Supplementary Files

File name: Supplementary Movie 1
Description: ‘Cyclic stretching of IHPS unit under a strain of 100% for 50 cycles’

File name: Supplementary Movie 2
Description: ‘Voltage test of IHPS unit under a strain of 100% for 1000 cycles’

File name: Supplementary Movie 3
Description: ‘Electrical performance of IHPS with 10 units in series under manual deformation’

File name: Supplementary Movie 4
Description: ‘Multimaterial printing process of IHPS at different switching frequencies’

File name: Supplementary Movie 5
Description: ‘Multimaterial printing process of IHPS at different switching frequencies’

File name: Supplementary Movie 6
Description: ‘Dissipation history of IHPS (unit proportion of 20:4:20:4) with 10 units in series for 100 h’

File name: Supplementary Movie 7
Description: ‘Automatically multimaterial printing and collection process of IHPS roll’

File name: Supplementary Movie 8
Description: ‘Automatically multimaterial printing and collection process of IHPS roll using a 16-arrayed multimaterial printhead’

File name: Supplementary Movie 9
Description: ‘Voltage measurement of IHPS with 1600 units in series’

File name: Supplementary Movie 10
Description: ‘Proof-of-concept for application of consecutively-printed IHPS’
